# A Newly Emerging HIV-1 Recombinant Lineage (CRF58_01B) Disseminating among People Who Inject Drugs in Malaysia

**DOI:** 10.1371/journal.pone.0085250

**Published:** 2014-01-22

**Authors:** Wei Zhen Chow, Yutaka Takebe, Nur Ezreen Syafina, Malarvelli Soorya Prakasa, Kok Gan Chan, Haider Abdulrazzaq Abed Al-Darraji, Clayton Koh, Adeeba Kamarulzaman, Kok Keng Tee

**Affiliations:** 1 Centre of Excellence for Research in AIDS (CERiA), Department of Medicine, Faculty of Medicine, University of Malaya, Kuala Lumpur, Malaysia; 2 AIDS Research Center, National Institute of Infectious Diseases, Toyama, Shinjuku-ku, Tokyo, Japan; 3 Division of Genetics and Molecular Biology, Institute of Biological Sciences, Faculty of Science, University of Malaya, Kuala Lumpur, Malaysia; University of Athens, Medical School, Greece

## Abstract

The HIV epidemic is primarily characterised by the circulation of HIV-1 group M (main) comprising of 11 subtypes and sub-subtypes (A1, A2, B–D, F1, F2, G, H, J, and K) and to date 55 circulating recombinant forms (CRFs). In Southeast Asia, active inter-subtype recombination involving three main circulating genotypes—subtype B (including subtype B′, the Thai variant of subtype B), CRF01_AE, and CRF33_01B—have contributed to the emergence of novel unique recombinant forms. In the present study, we conducted the molecular epidemiological surveillance of HIV-1 *gag*-RT genes among 258 people who inject drugs (PWIDs) in Kuala Lumpur, Malaysia, between 2009 and 2011 whereby a novel CRF candidate was recently identified. The near full-length genome sequences obtained from six epidemiologically unlinked individuals showed identical mosaic structures consisting of subtype B′ and CRF01_AE, with six unique recombination breakpoints in the *gag*-RT, *pol*, and *env* regions. Among the high-risk population of PWIDs in Malaysia, which was predominantly infected by CRF33_01B (>70%), CRF58_01B circulated at a low but significant prevalence (2.3%, 6/258). Interestingly, the CRF58_01B shared two unique recombination breakpoints with other established CRFs in the region: CRF33_01B, CRF48_01B, and CRF53_01B in the *gag* gene, and CRF15_01B (from Thailand) in the *env* gene. Extended Bayesian Markov chain Monte Carlo sampling analysis showed that CRF58_01B and other recently discovered CRFs were most likely to have originated in Malaysia, and that the recent spread of recombinant lineages in the country had little influence from neighbouring countries. The isolation, genetic characterization, and evolutionary features of CRF58_01B among PWIDs in Malaysia signify the increasingly complex HIV-1 diversity in Southeast Asia that may hold an implication on disease treatment, control, and prevention.

## Introduction

According to the Joint United Nations Program on HIV/AIDS (UNAIDS), approximately 34 million people were living with HIV worldwide by the end of 2011. Within the same year, 2.5 million new HIV infections were also reported across the globe, attributing to an adult HIV prevalence rate of 0.8% [1]. In Malaysia, a total of 94,841 cases of HIV infections had been reported since the country's first HIV epidemic began in 1986, among which 14,986 AIDS-related deaths were recorded. The high-risk practice of injecting drug use was especially prominent in Malaysia with the highest HIV prevalence rate at 70% compared to other risk groups and causing more than half of AIDS-related deaths in the country during the last two decades [2].

In Southeast Asia, the first HIV/AIDS epidemic occurred in Thailand in the late 1980s where two genetically distinct HIV-1 genotypes were co-circulating in the country, namely the circulating recombinant form (CRF) 01_AE (CRF01_AE) and subtype B (including subtype B′, the Thai variant of subtype B). However CRF01_AE and subtype B′ had circulated among distinct risk groups, where CRF01_AE propagated among those engaged in heterosexual activities as compared to subtype B′ circulating among people who inject drugs (PWIDs) [3], [4]. By mid-1990s, it was observed that the distribution of CRF01_AE was no longer confined among the heterosexuals when Tovanabutra et al. identified the circulation of CRF01_AE among 80% of PWID in Thailand [5]. Coupled with the rampant illegal drug trafficking activities in the region [6], CRF01_AE was soon disseminating among PWIDs in the vicinity including Cambodia, Vietnam, Malaysia, China, Taiwan, Korea, Japan and various countries in Southeast and East Asia [3], [7].

In the following years, in addition to the extensive genetic diversity of HIV-1 [8], the wide co-circulation and dual infection of CRF01_AE and subtype B′ among various risk populations in Southeast Asia have led to the emergence of various unique recombinant forms (URFs) and ultimately, CRFs as defined by the identification and characterisation of near full length HIV-1 sequences which display an identical mosaic genome isolated from three or more epidemiologically-unlinked persons [9]. At present, 55 CRFs have been characterised (http://www.hiv.lanl.gov/) and altogether they comprise an estimated 16% of HIV-1 infections reported worldwide [10]. In Southeast Asia, a recent study documented the massive expansion of CRF33_01B among PWIDs in Malaysia and its endemicity in various HIV-1 infected populations including children who acquired infections through their mothers – further highlighting the increasing transmission of CRF33_01B to the general population [11]. The CRF33_01B lineage is also reported to be actively recombining with the main circulating genotypes in the region, consequently generating multiple novel and genetically distinct clades including CRF48_01B and CRF53_01B [12], [13], each sharing one or more recombination features with CRF33_01B [14].

In addition to PWIDs, earlier studies reported the widespread dissemination of CRF33_01B at a significant prevalence among homosexuals and heterosexuals in Malaysia [14], [15] and also in neighbouring countries, in particular Singapore [16], [17], Indonesia [18] and Hong Kong [19], further demonstrating the establishment of the relatively new CRF33_01B lineages across Asia. The co-circulation of the previously identified CRFs and URFs, in addition to HIV-1 CRF01_AE, subtype B′ and other infrequent imported genotypes (e.g. subtype C, CRF02_AG [20]) may indeed increase the genetic complexity of HIV-1 in Southeast Asia. Furthermore, in view of the increasing epidemiological impact of HIV-1 recombinants, for example CRF33_01B [11] in Southeast Asian countries, it is highly presumptive that novel recombinants (CRF) could arise especially among the high risk injecting drug population. In this study, as a result of continuous molecular surveillance recently conducted among PWIDs between 2009 and 2011 in Malaysia [11], we report the emergence of a newly emerging novel HIV-1 CRF, designated as CRF58_01B characterised by the near full length recombinant genomes sequenced from six epidemiologically-unlinked PWIDs.

## Materials and Methods

### Ethics Statement

The study was approved by the University Malaya Medical Centre (UMMC) Medical Ethics Committee. Standard, multilingual consent forms allowed by the Medical Ethics Committee were used. Written consent was obtained from all willing study participants. Being an especially vulnerable population, all interviews and data collected were kept confidential. All potential participants who declined to participate in the study were not in any way disadvantaged from receiving treatment and care.

### Study Subjects and HIV-1 near Full Length Genome Amplification

All six study subjects were recruited during a molecular epidemiological study conducted during 2009–2011 among inmates of a prison and attendees of a needle syringe exchange program (n = 258) in Kuala Lumpur, Malaysia [11] based on initial HIV-1 *gag*-RT genes amplification and sequencing. Plasma samples were collected from all subjects, serologically determined to be HIV-1 positive and stored at −80°C until further processed.

HIV-1 Viral RNA was extracted from plasma samples using the NucliSENS easyMAG automated platform (bioMerieux, Durham, North Carolina, USA) according to the manufacturer's recommendation and reverse transcribed into cDNA using SuperScript III RNase H^−^ Reverse Transcriptase (Invitrogen, Carlsbad, California, USA) and random hexamers (Applied Biosystems, USA) according to the manufacturer's instructions. Nested PCR was performed using QIAGEN HotStarTaq *Plus* DNA polymerase (Qiagen, Hilden, Germany) to amplify 10 overlapping fragments corresponding to the near full length HIV-1 genome using primers listed in **Table S1**. Purified PCR products were directly sequenced using ABI PRISM 3730XL DNA Analyzer (Applied Biosystems, Foster City, California, USA) and assembled to produce near full length genomes (∼9 kb).

### Phylogenetic and Recombination Analysis

Near full length genomes were aligned with the HIV-1 reference subtypes and CRFs of epidemiological significance in Southeast Asia downloaded from the Los Alamos HIV database (http://www.hiv.lanl.gov/) using ClustalX 2.0. Nucleotide sequences were then manually adjusted using BioEdit 7.0 with reference to the HIV Sequence Compendium 2011 (http://www.hiv.lanl.gov/) to ensure accurate codon alignment. In addition, newly published CRFs of regional significance such as CRF48_01B and CRF51_01B to CRF55_01B were included in the alignment. Phylogenetic trees were constructed by the neighbour-joining method based on the Kimura two-parameter model with a transition-transversion ratio of 2.0 using MEGA 5.05 [21]. The reliability of the branching orders were analysed by bootstrap analysis of 1000 replicates. Bootscanning analysis [22] was performed using SimPlot version 3.5.1 [23] and followed by informative site analysis to identify the specific recombination breakpoints shared by the novel CRF candidates. All possible parental reference strains were included in the similarity plot to identify the closely related parental strains prior to subsequent analyses. Sub-region trees were constructed to confirm the parental origin of each segment in the near full length genome. Such methods have been established and widely used in various studies to characterize recombinant genomes, including HIV-1. All sequences reported in this study have been deposited in GenBank with accession numbers reported in **Table 1**.

**Table 1:** Epidemiological information of six non-epidemiologically linked study subjects infected with the novel CRF58_01B in Kuala Lumpur, Malaysia.

Sample ID	Age (years)	Sex	Ethnicity	Risk factors*	Sample collection (d/mo/yr)	Site of sample collection‡	Sequence length (bp)	Accession No.
09MYPR37	47	Male	Malay	PWID	01/10/2009	Prison	8975	KC522031
10MYKJ036	35	Male	Indian	PWID+Hetero	02/08/2010	Prison	8567	KC522035
10MYPR87	50	Male	Malay	PWID	15/04/2010	Prison	8931	KF425293
11MY1ZK731	38	Male	Malay	PWID	21/04/2011	NSEP	8934	KC522032
11MY1RJ704	41	Male	Malay	PWID	20/04/2011	NSEP	8914	KC522033
11MY1EP794	32	Male	Kadazan	PWID	23/04/2011	NSEP	8902	KC522034

PWID indicates people who inject drugs; Hetero, Heterosexual.

NSEP; needle syringe exchange program.

## Results and Discussions

### Phylogenetic and Recombination Analysis Identified a Novel HIV-1 CRF58_01B Genotype

**Figure 1:**
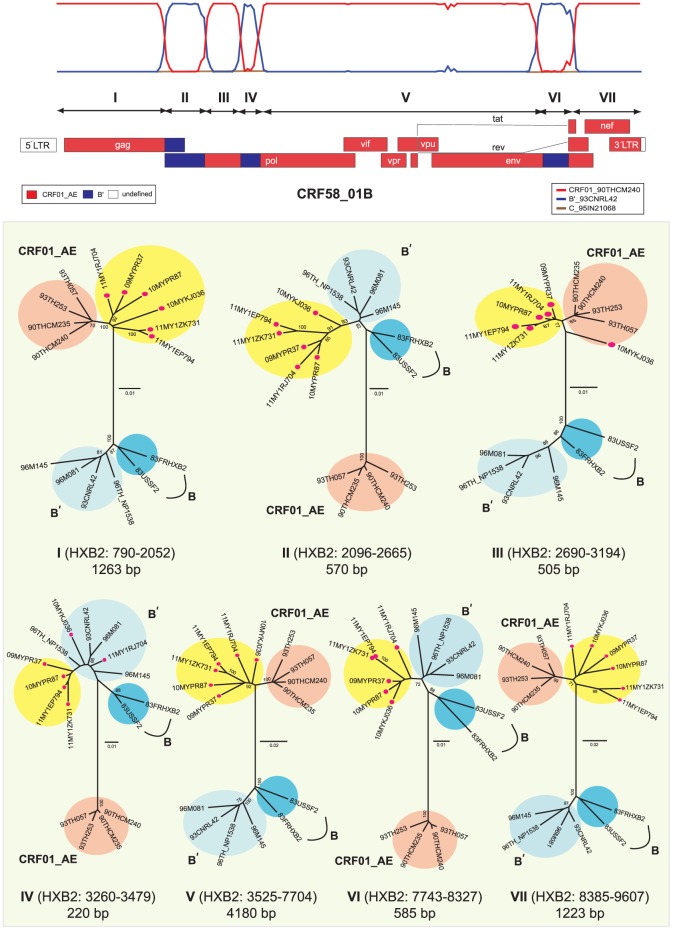
The near full length mosaic structure of HIV-1 CRF58_01B determined using bootscanning and informative site analysis. CRF58_01B composed of three subtype B′ fragments recombined with CRF01_AE in the *gag-*RT, *pol* and *env* regions of HIV-1. Analysis revealed the sharing of similar unique mosaic structures and recombination breakpoints between the six strains (09MYPR37, 10MYKJ036, 10MYPR87, 11MY1ZK731, 11MY1RJ704 and 11MY1EP794), thus constituting a novel CRF58_01B genotype. HIV-1 reference strains CRF01_CM240 (CRF01_AE) and B′_RL42 (Thai subtype B′) were selected as the putative parental genotypes by similarity plotting, and C_95IN21068 (subtype C) as outgroup, with a window size of 400 nucleotides moving along the alignment in increments of 50 nucleotides to define the recombination structures. Sub-region neighbour-joining trees were constructed in MEGA 5.05 using Kimura 2-parameter method for nucleotide substitutions to estimate pair-wise evolutionary distance. The reliability of the branch nodes were assessed by bootstrap analysis of 1000 replicates. Bootstrap values of greater than 70% were indicated on the branch nodes. The scale bar of the individual sub-region trees were indicated in substitutions per site.

In the present study, we sequenced the near full length genomes of HIV-1 isolated from six epidemiologically unlinked PWIDs recruited as part of a recent molecular epidemiological surveillance conducted between 2009 and 2011 in Kuala Lumpur, Malaysia. These strains were selected for near full length sequencing based on the preliminary screening of the *gag*-RT genes which revealed the sharing of unique recombination structures and breakpoints [11]. All subjects were antiretroviral-naïve males with a mean age of 40.5±6.9 years old from various ethnic groups (Malays, Indian and Kadazan) with a history of unsafe injecting drug use (**Table 1**). In these six strains (09MYPR37, 10MYKJ036, 10MYPR87, 11MY1ZK731, 11MY1RJ704 and 11MY1EP794), the coding regions of HIV-1 were amplified, spanning the *gag*, *pol*, *env*, *tat*, *rev*, *vif*, *vpr*, *vpu*, and *nef* genes and subsequently analysed for genetic evidence of similar mosaic recombination structures using SimPlot (bootscanning and informative sites analyses). Bootscanning and informative sites analyses of the near full length genomes were performed using HIV-1 CRF01_CM240 and B′_RL42 as the putative parental genotypes, revealing similar mosaic recombination structures in all six strains (**Figure 1** **and Table S2**). In these mosaic genomes involving HIV-1 CRF01_AE and subtype B′, CRF01_AE regions were identified at HXB2 positions 790 to 2052, 2690 to 3194, 3525 to 7704 and 8385 to 9607 nt.

**Figure 2:**
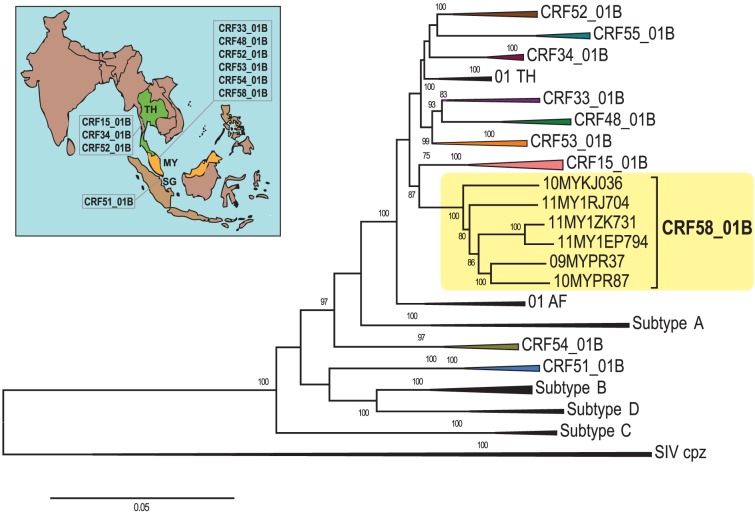
Phylogenetic reconstruction of near full length HIV-1 circulating recombinant forms (CRFs) in Southeast Asia. Near full length genomes of CRFs discovered in the region, including the newly characterised CRF58_01B among people who inject drugs (PWIDs) in Kuala Lumpur, Malaysia were analysed. Neighbour-joining tree was constructed in MEGA 5.05 using the Kimura 2-parameter method of nucleotide substitutions to estimate pair-wise evolutionary distance and the reliability of the branching nodes were assessed by bootstrap analysis of 1000 replicates. Reference strains of established and informative HIV-1 genotypes – CRF01_AE, subtype B (including Thai B′), CRF15_01B, CRF34_01B, CRF33_01B, CRF48_01B, CRF51_01B, CRF52_01B, CRF53_01B, CRF54_01B and CRF55_01B (from China) were included in the analysis. Other HIV-1 genotypes of group M – subtypes A, C and D were also included to depict the diverse genetic diversity of HIV-1 with SIVcpz reference strains as outgroup. Of note, CRF58_01B forms a strongly supported clade being distinct from other established genotypes. Bootstrap values of greater than 70% were indicated on the branch nodes. The scale bar represents 5% genetic distance (0.05 substitutions per site).

Three short subtype B′ fragments were present in the *gag*-RT, *pol* and *env* regions (HXB2 positions 2096 to 2665, 3260 to 3479 and 7743 to 8327 nt) with unique recombination breakpoints estimated at HXB2 positions 2053 to 2095 and 2666 to 2689; 3195 to 3259 and 3480 to 3524; 7705 to 7742 and 8328 to 8384 nt, respectively. Sub-region neighbour joining tree analyses confirmed the parental origin of each region (designated as region I to VII) of the mosaic near full length recombinant genomes (**Figure 1**). Regions I, III, V and VII (1263 bp, 505 bp, 4180 bp and 1223 bp, consecutively) were grouped within CRF01_AE and regions II, IV and VI (570 bp, 220 bp and 585 bp, consecutively) were of subtype B′ origin. Of interest, the first recombination breakpoint (HXB2: 2053 to 2095 nt) shared among all six strains was similarly identified in CRF33_01B [14], [24], CRF48_01B [12] and CRF53_01B [13], firstly identified in Malaysia. Moreover, the sixth unique recombination breakpoint of all six strains was located closely with that of CRF15_01B, originally identified in Thailand [25] in the *env* region (approximately 12 bp in distance relative to the recombination breakpoint of CRF15_01B, reported at position 8317±1 nt by the Los Alamos National Laboratory, LANL). Taken together, the near full length genomes of all six strains formed a highly-supported novel cluster, genetically distinct from other established subtypes and CRFs reported worldwide and therefore assigned as CRF58_01B by the LANL, in compliance with the HIV-1 nomenclature system (**Figure 2**). Intra-genotype pairwise nucleotide distance analysis of CRF58_01B and its parental strains (CRF01_AE and B/B′) based on a larger alignment data set containing CRF01_AE (n = 59) and B/B′ (n = 77) reference sequences has shown that both parental strains had greater nucleotide diversity across the genomes compared to CRF58_01B, although the estimated nucleotide diversity within CRF58_01B sequences was considerably high **(Table S2)**. This may imply that CRF58_01B could have recombined years ago and not recently. There are perhaps several reasons why CRF58_01B was not discovered during the early years: a) CRF58_01B was circulating at a very low level and concentrated among the PWIDs, b) insufficient sampling in previous molecular epidemiology studies, and c) mis-classification of CRF58_01B due to limited, partial genome analysis (often only involving the *prot*-RT genes).

### Evolutionary Analysis of CRF58_01B and other Established CRFs in Malaysia and Thailand

**Figure 3:**
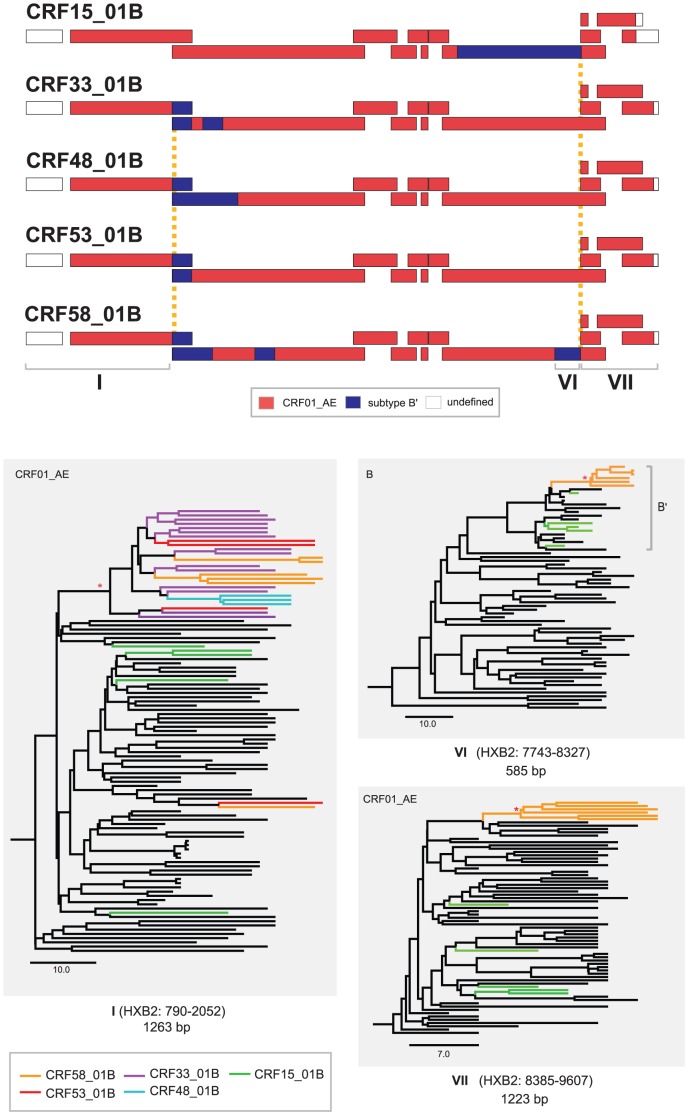
Near full length mosaic genomes of circulating recombinant form, CRF58_01B and other established CRFs in Malaysia and Thailand. Recently isolated among PWIDs in Malaysia, CRF58_01B shared two unique recombination breakpoints (indicated in dashed lines) with other established CRFs in the region: CRF33_01B, CRF48_01B and CRF53_01B in the *gag* gene at HXB2: 2053 to 2095 nt, and CRF15_01B (from Thailand) in the *env* gene. Maximum clade credibility (MCC) phylogenies were constructed for the shared sub-regions I, VI and VII between CRF58_01B and the established CRFs by including CRF01_AE (sampled from 1990 to 2009 in Malaysia, Thailand, China and Japan) and B/B′ reference strains (sampled from 1983 to 2011 in France, United States of America, Japan, Thailand and Myanmar) downloaded from the Los Alamos HIV database (labelled in black) to discern their evolutionary relationship. Monophyletic clusters (with a posterior probability of 1.0) were indicated with an asterisk (*) at the branch nodes. SIVcpz and CRF01_AE or B/B′ reference strains were included as outgroups but not shown for simplicity. The sub-region trees were scaled in units of time (years).

Following the near full length genome characterization of HIV-1 CRF58_01B, we further distinguished the probable genetic relationships between CRF58_01B and other established CRFs in Malaysia and Thailand on the basis of the two shared unique recombination breakpoints with CRF33_01B, CRF48_01B and CRF53_01B in the *gag* gene, and CRF15_01B in the *env* gene. We hypothesize that CRF58_01B may be ancestrally linked to the established CRFs mentioned herein, therefore phylogenetic signal within genetic regions adjacent to the breakpoints may possibly reveal the shared evolutionary history among these lineages [26]. Briefly, maximum clade credibility (MCC) phylogenetic reconstructions were performed for the three shared sub-regions: region I (of CRF01_AE origin, HXB2: 790–2052 nt), VI (subtype B′ origin, HXB2: 7743–8327 nt) and VII (CRF01_AE origin, HXB2: 8385–9607 nt) in the *gag*, *env* and *nef-*3′ LTR genes, respectively (**Figure 3**). Reference strains of CRF01_AE (sampled from 1990 to 2009 in Malaysia, Thailand, China and Japan) and B/B′ (sampled from 1983 to 2011 in France, United States of America, Japan, Thailand and Myanmar) were downloaded from the Los Alamos HIV database. In addition, reference strains of CRF15_01B (n = 5), CRF33_01B (n = 15), CRF48_01B (n = 3), CRF53_01B (n = 4) and the putative parental genotypes of these recombinants, CRF01_AE and subtype B (including B′) from Southeast and East Asia, were also retrieved from the online database. A coalescent-based Bayesian Markov chain Monte Carlo (MCMC) sampling method was performed in BEAST v1.7.4 [27] with an uncorrelated lognormal relaxed molecular clock and appropriate evolutionary parameters incorporated, as described previously [26].

First, MCC phylogeny analysis of the *gag* gene (region I) showed that almost all CRFs isolated from Malaysia (CRF33_01B, CRF48_01B, CRF53_01B and the novel CRF58_01B) were intermingled within a robust monophyletic cluster distinct from other CRF01_AE and CRF15_01B strains (**Figure 3**). This could possibly be explained by the high rates of recombination that may confound the evolutionary history and classification of HIV-1 sequences [28]. Recombination event especially intra-subtype recombination (recombination involving closely related lineages of the same subtype within a single individual) is common among HIV-1. Such mechanism could lead to possible discrepancies on linkage disequilibrium, including the possible loss of phylogenetic correlation within the genome. Most of the analysed CRFs (CRF33/48/53/58) were likely to have emerged some years ago as indicated by the high intra-subtype genetic distances **(** **Figure 3** **)**, thus increasing the likelihood of intra-subtype recombination that may lead to such discrepancy [29]. Such genetic pattern indicated that a common ancestor was shared among these CRFs, and recombination events that generated these clades were likely to be traced to Malaysia. In addition, the findings probably showed that recent emergence of novel CRFs, which is thought to be the major driving force of the regional epidemic, have had little influence from neighbouring countries. The spatial and temporal structure in the *gag* gene however was less informative to elucidate clearly the divergence times for each CRF lineage. Lastly, in order to examine the evolutionary relationship between CRF58_01B and CRF15_01B (mainly circulating in Thailand), MCC tree analysis for sub-regions VI and VII showed that all CRF58_01B strains were grouped together but distantly located from all CRF15_01B sequences (**Figure 3**). The results disproved the possible genealogical relationship between CRF58_01B and CRF15_01B, although both genotypes shared a common recombination “hotspot” in the *env* gene.

The increasing genetic diversity of HIV-1 in Southeast Asia was attributed to the growing emergence of distinct CRFs circulating in the HIV-1 infected population in the region. In recent years (2005–2011), a total of five novel and genetically distinct CRFs, namely CRF33_01B [14], [24], CRF48_01B [12], CRF52_01B [30], CRF53_01B [13] and CRF54_01B [31] (**Figure 2**) had been identified among various HIV-1 infected populations, mostly involving PWIDs, heterosexuals and men who have sex with men (MSM) in Malaysia whereby active inter-subtype recombination between subtype B′, CRF01_AE and CRF33_01B lineages has been on-going. The identification of CRF58_01B may represent one of the recently emerging recombinant strains circulating at a low prevalence (2.3%, 6/258) among PWIDs in the region, as indicated by its apparent absence in other molecular epidemiological studies conducted prior to year 2009 [14], [24], [32]–[35]. The steady emergence of CRF58_01B between 2009 and 2011 however was not readily explained and remains unclear, although it is possible that the increased detection of CRF58_01B especially in year 2011 may be due to active transmission among PWIDs.

In summary, the present study identified a newly emerging HIV-1 CRF58_01B among PWIDs in Malaysia and in addition to other co-circulating genotypes adds further challenges to the development of an HIV vaccine and HIV-1 control in general.

## Supporting Information

Table S1 HIV-1 near full length primer sequences (in 5′ to 3′ direction).
(DOCX)

Table S2 Intra-genotype pairwise nucleotide distances of CRF58_01B and its putative parental reference strains (CRF01_AE and B/B′) (supplementary of Figure 1).
(DOCX)

## References

[1] Joint United Nations Programme on HIV/AIDS (UNAIDS) (2012) Global Report: UNAIDS Report On The Global AIDS Epidemic 2012.

[2] Ministry of Health (2012) Global AIDS Response Country Progress Report 2012: Malaysia.

[3] Weniger BG, Takebe Y, Ou CY, Yamazaki S (1994) The molecular epidemiology of HIV in Asia. AIDS 8 Suppl 2: S13–28. 7857556

[4] Ou CY, Takebe Y, Weniger BG, Luo CC, Kalish ML, et al (1993) Independent introduction of two major HIV-1 genotypes into distinct high-risk populations in Thailand. Lancet 341: 1171–1174. 8098076 10.1016/0140-6736(93)91001-3

[5] Tovanabutra S, Polonis V, De Souza M, Trichavaroj R, Chanbancherd P, et al (2001) First CRF01_AE/B recombinant of HIV-1 is found in Thailand. AIDS 15: 1063–1065. 11399992 10.1097/00002030-200105250-00018

[6] Beyrer C, Razak MH, Lisam K, Chen J, Lui W, et al (2000) Overland heroin trafficking routes and HIV-1 spread in south and south-east Asia. AIDS 14: 75–83. 10714570 10.1097/00002030-200001070-00009

[7] Weniger BG, Brown T (1996) The march of AIDS through Asia. N Engl J Med 335: 343–345. 8663856 10.1056/NEJM199608013350510

[8] Preston BD, Poiesz BJ, Loeb LA (1988) Fidelity of HIV-1 reverse transcriptase. Science 242: 1168–1171. 2460924 10.1126/science.2460924

[9] Robertson DL, Anderson JP, Bradac JA, Carr JK, Foley B, et al (2000) HIV-1 nomenclature proposal. Science 288: 55–56. 10766634 10.1126/science.288.5463.55d

[10] Hemelaar J, Gouws E, Ghys PD, Osmanov S (2011) Global trends in molecular epidemiology of HIV-1 during 2000–2007. AIDS 25: 679–689. 21297424 10.1097/QAD.0b013e328342ff93 PMC3755761

[11] Chow WZ, Ong LY, Razak SH, Lee YM, Ng KT, et al (2013) Molecular diversity of HIV-1 among people who inject drugs in Kuala Lumpur, Malaysia: massive expansion of circulating recombinant form (CRF) 33_01B and emergence of multiple unique recombinant clusters. PLoS One 8: e62560. 23667490 10.1371/journal.pone.0062560 PMC3646884

[12] Li Y, Tee KK, Liao H, Hase S, Uenishi R, et al (2010) Identification of a novel second-generation circulating recombinant form (CRF48_01B) in Malaysia: a descendant of the previously identified CRF33_01B. J Acquir Immune Defic Syndr 54: 129–136. 20386110 10.1097/QAI.0b013e3181d82ce5

[13] Chow WZ, Al-Darraji H, Lee YM, Takebe Y, Kamarulzaman A, et al (2012) Genome sequences of a novel HIV-1 CRF53_01B identified in Malaysia. J Virol 86: 11398–11399. 22997419 10.1128/JVI.01932-12 PMC3457147

[14] Tee KK, Li XJ, Nohtomi K, Ng KP, Kamarulzaman A, et al (2006) Identification of a novel circulating recombinant form (CRF33_01B) disseminating widely among various risk populations in Kuala Lumpur, Malaysia. J Acquir Immune Defic Syndr 43: 523–529. 17031320 10.1097/01.qai.0000242451.74779.a7

[15] Wang B, Lau KA, Ong LY, Shah M, Steain MC, et al (2007) Complex patterns of the HIV-1 epidemic in Kuala Lumpur, Malaysia: evidence for expansion of circulating recombinant form CRF33_01B and detection of multiple other recombinants. Virology 367: 288–297. 17604072 10.1016/j.virol.2007.05.033

[16] Lee CC, Sun YJ, Barkham T, Leo YS (2009) Primary drug resistance and transmission analysis of HIV-1 in acute and recent drug-naive seroconverters in Singapore. HIV Med 10: 370–377. 19490177 10.1111/j.1468-1293.2009.00698.x

[17] Ng OT, Lin L, Laeyendecker O, Quinn TC, Sun YJ, et al (2011) Increased rate of CD4+ T-cell decline and faster time to antiretroviral therapy in HIV-1 subtype CRF01_AE infected seroconverters in Singapore. PLoS One 6: e15738. 21298051 10.1371/journal.pone.0015738 PMC3029292

[18] SahBandar IN, Takahashi K, Motomura K, Djoerban Z, Firmansyah I, et al (2011) The indonesian variants of CRF33_01B: Near-full length sequence analysis. AIDS Res Hum Retroviruses 27: 97–102. 20958201 10.1089/aid.2010.0163 PMC5586156

[19] Chen JH, Wong KH, Chen Z, Chan K, Lam HY, et al (2010) Increased genetic diversity of HIV-1 circulating in Hong Kong. PLoS One 5: e12198. 20808942 10.1371/journal.pone.0012198 PMC2922374

[20] Ong LY, Razak SH, Lee YM, Ponnampalavanar SSLS, Omar SFS, et al (2013) Molecular Diversity of HIV-1 and Surveillance of Transmitted Drug Resistance Variants among Treatment-naïve Patients, 5 years after Active Introduction of HAART in Kuala Lumpur, Malaysia. Journal of Medical Virology 86 1: 38–44. 24127302 10.1002/jmv.23772

[21] Tamura K, Peterson D, Peterson N, Stecher G, Nei M, et al (2011) MEGA5: molecular evolutionary genetics analysis using maximum likelihood, evolutionary distance, and maximum parsimony methods. Mol Biol Evol 28: 2731–2739. 21546353 10.1093/molbev/msr121 PMC3203626

[22] Salminen MO, Carr JK, Burke DS, McCutchan FE (1995) Identification of breakpoints in intergenotypic recombinants of HIV type 1 by bootscanning. AIDS Res Hum Retroviruses 11: 1423–1425. 8573403 10.1089/aid.1995.11.1423

[23] Lole KS, Bollinger RC, Paranjape RS, Gadkari D, Kulkarni SS, et al (1999) Full-length human immunodeficiency virus type 1 genomes from subtype C-infected seroconverters in India, with evidence of intersubtype recombination. J Virol 73: 152–160. 9847317 10.1128/jvi.73.1.152-160.1999 PMC103818

[24] Tee KK, Pon CK, Kamarulzaman A, Ng KP (2005) Emergence of HIV-1 CRF01_AE/B unique recombinant forms in Kuala Lumpur, Malaysia. AIDS 19: 119–126. 15668536 10.1097/00002030-200501280-00003

[25] Tovanabutra S, Watanaveeradej V, Viputtikul K, De Souza M, Razak MH, et al (2003) A new circulating recombinant form, CRF15_01B, reinforces the linkage between IDU and heterosexual epidemics in Thailand. AIDS Res Hum Retroviruses 19: 561–567. 12908933 10.1089/088922203322230923

[26] Tee KK, Pybus OG, Li XJ, Han X, Shang H, et al (2008) Temporal and spatial dynamics of human immunodeficiency virus type 1 circulating recombinant forms 08_BC and 07_BC in Asia. J Virol 82: 9206–9215. 18596096 10.1128/JVI.00399-08 PMC2546895

[27] Drummond AJ, Suchard MA, Xie D, Rambaut A (2012) Bayesian Phylogenetics with BEAUti and the BEAST 1.7. Mol Biol Evol 29: 1969–1973. 22367748 10.1093/molbev/mss075 PMC3408070

[28] Abecasis AB, Lemey P, Vidal N, de Oliveira T, Peeters M, et al (2007) Recombination confounds the early evolutionary history of human immunodeficiency virus type 1: subtype G is a circulating recombinant form. J Virol 81: 8543–8551. 17553886 10.1128/JVI.00463-07 PMC1951349

[29] Schierup MH, Hein J (2000) Consequences of recombination on traditional phylogenetic analysis. Genetics 156: 879–891. 11014833 10.1093/genetics/156.2.879 PMC1461297

[30] Liu Y, Li L, Bao Z, Li H, Zhuang D, et al (2012) Identification of a novel HIV type 1 circulating recombinant form (CRF52_01B) in Southeast Asia. AIDS Res Hum Retroviruses 28: 1357–1361. 22269007 10.1089/aid.2011.0376 PMC3448130

[31] Ng KT, Ong LY, Takebe Y, Kamarulzaman A, Tee KK (2012) Genome sequence of a novel HIV-1 circulating recombinant form 54_01B from Malaysia. J Virol 86: 11405–11406. 22997423 10.1128/JVI.01949-12 PMC3457179

[32] Tee KK, Saw TL, Pon CK, Kamarulzaman A, Ng KP (2005) The evolving molecular epidemiology of HIV type 1 among injecting drug users (IDUs) in Malaysia. AIDS Res Hum Retroviruses 21: 1046–1050. 16379608 10.1089/aid.2005.21.1046

[33] Saraswathy TS, Ng KP, Sinniah M (2000) Human immunodeficiency virus type 1 subtypes among Malaysian intravenous drug users. Southeast Asian J Trop Med Public Health 31: 283–286. 11127327

[34] Beyrer C, Vancott TC, Peng NK, Artenstein A, Duriasamy G, et al (1998) HIV type 1 subtypes in Malaysia, determined with serologic assays: 1992–1996. AIDS Res Hum Retroviruses 14: 1687–1691. 9870323 10.1089/aid.1998.14.1687

[35] Brown TM, Robbins KE, Sinniah M, Saraswathy TS, Lee V, et al (1996) HIV type 1 subtypes in Malaysia include B, C, and E. AIDS Res Hum Retroviruses 12: 1655–1657. 8947304 10.1089/aid.1996.12.1655

