# Supplementary material for: A Newly Emerging HIV-1 Recombinant Lineage (CRF58_01B) Disseminating among People Who Inject Drugs in Malaysia
Source: PLoS One. 2014 Jan 22;9(1):e85250. doi: 10.1371/journal.pone.0085250 (PMC3898983; doi:10.1371/journal.pone.0085250)
Supplement: Table S1 — HIV-1 near full length primer sequences (in 5′ to 3′ direction). (DOCX) [file pone.0085250.s001.docx]

| **Region**  **Table S1. HIV-1 near full length primer sequences (in 5' to 3' direction).** | **Primer name** | **Primer Sequence** | **Location in HXB2 (nt)** |
| --- | --- | --- | --- |
| 1 (*gag*) | 172 A | ATCTCTAGCAGTGGCGCCCGAACAG | 625-649 |
|  | 505 B | ACTCTTGCTTTATGGCTGGGTCC | 1874-1852 |
|  | 174A | CTCTCGACGCAGGACTCGGCTTGCT | 683-707 |
|  | 506B | CCTGACATGCTGTCATCATTTCTTCTA | 1843-1817 |
| 2 (*prot*) | 507A | AAGGAACCCTTTAGAGACTATGTAGA | 1657-1682 |
|  | 503B | TATGGATTTTCAGGCCCAATTTTTG | 2692-2716 |
|  | 508A | GTAAAAAATTGGATGACAGAAACCTTG | 1726-1752 |
|  | 504B | ACTTTTGGGCCATCCATTCC | 2611-2592 |
| 3 (RT) | K1 | GGAAACCAAAAATGATAGGGGGAATTGGAGG | 2377 - 2407 |
|  | K2 (326B) | CTGTACTTCTGCTACTAAGTCTTTTGATGGG | 3539 - 3509 |
|  | K3 | GTGGAAAAAAGGCTATAGGTACAG | 2452 - 2475 |
|  | K4 (328B) | CTGCCAACTCTAATTCTGCTTC | 3462 - 3441 |
| 4 (*pol*) | polE-301A | TACACCAGACAAAAAGCATCAG | 3194 - 3215 |
|  | AGRNB-346 | TCTACTTGTTCATTTCCTCC | 4176 - 4193 |
|  | pol-461A | GGCAACTTTGTAAACTCCTTAGGGGG | 3379 - 3404 |
|  | pol-455B | CCCTCCAAT TCCTTTGTGTGCTGGC | 4157 - 4181 |
| 5 (*int*) | pol-491A | CTATGTAGATGGGGCAGCTA | 3869 - 3888 |
|  | int-308B | TACTGCCCCTTCACCTTTCCA | 4956 – 4976 |
|  | pol-485A | AAGTAAACATAGTAACAGACTCAC | 4024 - 4047 |
|  | int-310B | GCTGTCCCTGTAATAAACCCG | 4899 - 4919 |
| 6 (*vif*) | int-309A | TAAGACAGCAGTACAAATGGCAG | 4745 - 4767 |
| **Region** | **Primer name** | **Primer Sequence** | **Location in HXB2 (nt)** |
|  | vpr1B-364B | GGCTGACTTCCTGGATGGTTCCAGGGC | 5858 - 5884 |
|  | pol-465A | GGGGGGATTGGGGGGTACAGTGCAGGGG | 4794 - 4821 |
|  | TATCM-144B | AGGGCTCTAGGTTAGGATCTACCAGTTCCA | 5833 - 5862 |
| 7 (*vpu*) | vpr-463A | TTCATTTCAGAATTGGGTG | 5767 - 5786 |
|  | gp120-538B | CTAGACTACCATTTAACAGCA | 7000 - 7020 |
|  | VPRE-081Af | TTGGGTGTCAACATAGCAGAATAGG | 5779 - 5803 |
|  | C2-447B | AGTTGAGTTGATACCACTGGC | 7000 - 6980 |
| 8 (*env*) | ENV A (C2V5-F1) | CTCCAGCTGGTTWTGCRATT | 6880-6899 |
|  | ENV B (TM-004B) | GTCTGGCCTGTACCGTCAGCG | 7851-7831 |
|  | ENV C (C2V5-F2) | CAGCTGGTTWTGCGATTCTAA | 6883-6903 |
|  | ENV D (ENV-521B) | GCCCATAGTGCTTCCTGCTGCTCC | 7817-7794 |
| 9 (gp41) | gp160-545A | GACAAT TGGAGAAGTGAATT | 7653 - 7672 |
|  | gp41-537B | AGACACTGCTCCTACTCCTTCTG | 8876 - 8898 |
|  | env-458A | GGGCAAAGAGAAGAGTGGTGG | 7723 - 7743 |
|  | NEF-005B | TTTGACCACTTGCCACCCAT | 8797 - 8816 |
| 10 (*nef*-3'LTR) | Env43F14 | GAGTTAGGCAGGGATACTTCAC | 8344 - 8364 |
|  | 3'LTR43R16 | TAAGCACTCAAGGCAAGC | 9618 - 9635 |
|  | Env43F15 | AGCCTGTGCCTCTTCAGCTACCA | 8508 - 8530 |
|  | MSR5 | GCACTCAAGGCAAGC TTTATTGAGGCT | 547 - 521, 9606 - 9632 |
