# Supplementary material for: A Newly Emerging HIV-1 Recombinant Lineage (CRF58_01B) Disseminating among People Who Inject Drugs in Malaysia
Source: PLoS One. 2014 Jan 22;9(1):e85250. doi: 10.1371/journal.pone.0085250 (PMC3898983; doi:10.1371/journal.pone.0085250)
Supplement: Table S2 — Intra-genotype pairwise nucleotide distances of CRF58_01B and its putative parental reference strains (CRF01_AE and B/B′) (supplementary of Figure 1). (DOCX) [file pone.0085250.s002.docx]

**Table S2. Intra-genotype pairwise nucleotide distances of CRF58_01B and its putative parental reference strains (CRF01_AE and B/B') (supplementary of Figure 1)**

| HIV-1 genotypes/ Sub-region trees | I  (*gag*) | II  (*pol*) | III  (*pol*) | IV  (*pol*) | V  (*pol*-*env*) | VI  (*env*) | VII  (*env-*3' LTR) |
| --- | --- | --- | --- | --- | --- | --- | --- |
| 1. CRF01_AE | 0.030±0.002 |  | 0.037±0.003 |  | 0.064±0.002 |  | 0.071±0.005 |
| 2. B/B' |  | 0.057±0.004 |  | 0.069±0.008 |  | 0.077±0.006 |  |
| 3. CRF58_01B | 0.029±0.003 | 0.035±0.005 | 0.019±0.004 | 0.038±0.009 | 0.053±0.003 | 0.027±0.005 | 0.070±0.006 |
